# Automated smartphone-based system for measuring sperm viability, DNA fragmentation, and hyaluronic binding assay score

**DOI:** 10.1371/journal.pone.0212562

**Published:** 2019-03-13

**Authors:** Irene Dimitriadis, Charles L. Bormann, Manoj Kumar Kanakasabapathy, Prudhvi Thirumalaraju, Hemanth Kandula, Vinish Yogesh, Neeraj Gudipati, Vignesh Natarajan, John C. Petrozza, Hadi Shafiee

**Affiliations:** 1 Department of Obstetrics & Gynecology, Division of Reproductive Endocrinology and Infertility, Massachusetts General Hospital, Harvard Medical School, Boston, Massachusetts, United States of America; 2 Department of Medicine, Division of Engineering in Medicine, Brigham and Women’s Hospital, Harvard Medical School, Boston, Massachusetts, United States of America; 3 Department of Medicine, Harvard Medical School, Boston, Massachusetts, United States of America; Texas A&M University College Station, UNITED STATES

## Abstract

The fundamental test for male infertility, semen analysis, is mostly a manually performed subjective and time-consuming process and the use of automated systems has been cost prohibitive. We have previously developed an inexpensive smartphone-based system for at-home male infertility screening through automatic and rapid measurement of sperm concentration and motility. Here, we assessed the feasibility of using a similar smartphone-based system for laboratory use in measuring: a) Hyaluronan Binding Assay (HBA) score, a quantitative score describing the sperm maturity and fertilization potential in a semen sample, b) sperm viability, which assesses sperm membrane integrity, and c) sperm DNA fragmentation that assesses the degree of DNA damage. There was good correlation between the manual analysis and smartphone-based analysis for the HBA score when the device was tested with 31 fresh, unprocessed human semen samples. The smartphone-based approach performed with an accuracy of 87% in sperm classification when the HBA score was set at manufacturer’s threshold of 80. Similarly, the sperm viability and DNA fragmentation tests were also shown to be compatible with the smartphone-based system when tested with 102 and 47 human semen samples, respectively.

## Introduction

Infertility estimates suggest that worldwide, almost 50 million couples are affected and the male factor has been reported as the cause for 20% to 70% of these cases [1]. *Agarwal et al*. showed that up to 12% of men will suffer from infertility during their lifetime, with rates in Africa and Eastern Europe being the highest [2]. Semen analysis has always been the primary test for male infertility. Advances in consumer electronics and microfabrication and mobile health solutions can help us in developing new innovative tools for rapid, low-cost, automated semen analysis at the point-of-care. In an earlier work we have shown, that using an optical attachment, a smartphone can be used to accurately measure sperm concentration and motility [3]. Although sperm concentration, motility, and morphology are the basic components for determining specimen quality, other laboratory tests have been developed which provide information regarding the functionality of spermatozoa in cases of male factor infertility [4]. Among these are the (i) Hyaluronic Binding Assay (HBA), (ii) sperm viability test, and (iii) sperm DNA fragmentation test.

The HBA test is used to assess a semen sample to quantitatively determine the overall sperm maturity status and fertilization potential [5]. HBA score can potentially be used as a valuable parameter along with sperm concentration and motility for an enhanced male infertility diagnosis. Currently, HBA analysis is labor-intensive as it is performed manually using a laboratory-based microscope.

For normal fertility, approximately 60% or more of a male’s sperm in a fresh ejaculate of semen sample should be viable. Sperm motility can be used to approximate the proportion of viable sperm in a semen sample. However, to distinguish immotile live sperm from dead sperm, a sperm viability test is required. There are many methods of assessing sperm viability, most of which are based on the ability of the cell membrane to exclude dyes from entering the sperm and permeating into the nucleus [6, 7]. While there are a few techniques available for clinical use, the eosin-nigrosin staining-based approach for sperm viability testing is currently one of the most commonly used methods.

Lastly, DNA damage present in a given sperm population is measured using DNA fragmentation assays. Sperm DNA fragmentation has been correlated with both sperm motility and morphology [4, 8, 9]. The Halosperm kit is a sperm chromatin dispersion test that is available for routine clinical assessments of the human sperm DNA fragmentation and is one of the most commonly used methods owing to its accuracy and simplicity [10].

Advances in smartphone technologies and software have revolutionized the field of biomedical engineering. Over 100 software applications (apps) are currently available on smartphones that are of use to the biomedical research community. The ability of these applications is greatly augmented by peripheral devices that provide additional capabilities to the smartphone hardware. One such hardware is the portable optical attachment that effectively turns a smartphone into a microscope. It has been shown that integrating microfluidics, nanotechnology, and smartphone-based optical sensing can help in developing mobile health diagnostics with applications in managing infectious diseases and infertility [3, 11–17].

Here, we sought to improve our smartphone-based automated system, already proven to, rapidly and accurately, measure sperm concentration and motility to identify abnormal samples as defined by the 2010 WHO guidelines in unwashed, undiluted semen samples [3, 18], by evaluating its ability to provide information on HBA score, sperm viability, and sperm DNA fragmentation.

## Materials and methods

### Study design

Prior to the initiation of this study, the use of discarded specimens was approved by the Human Studies Institutional Review Boards of the Massachusetts General Hospital (MGH). HBA scores, sperm viability and sperm DNA fragmentation were all assessed separately. All three sperm tests were measured with the smartphone system and their results were compared to those obtained through traditional manual analysis.

### Optical attachment

**Fig 1:**
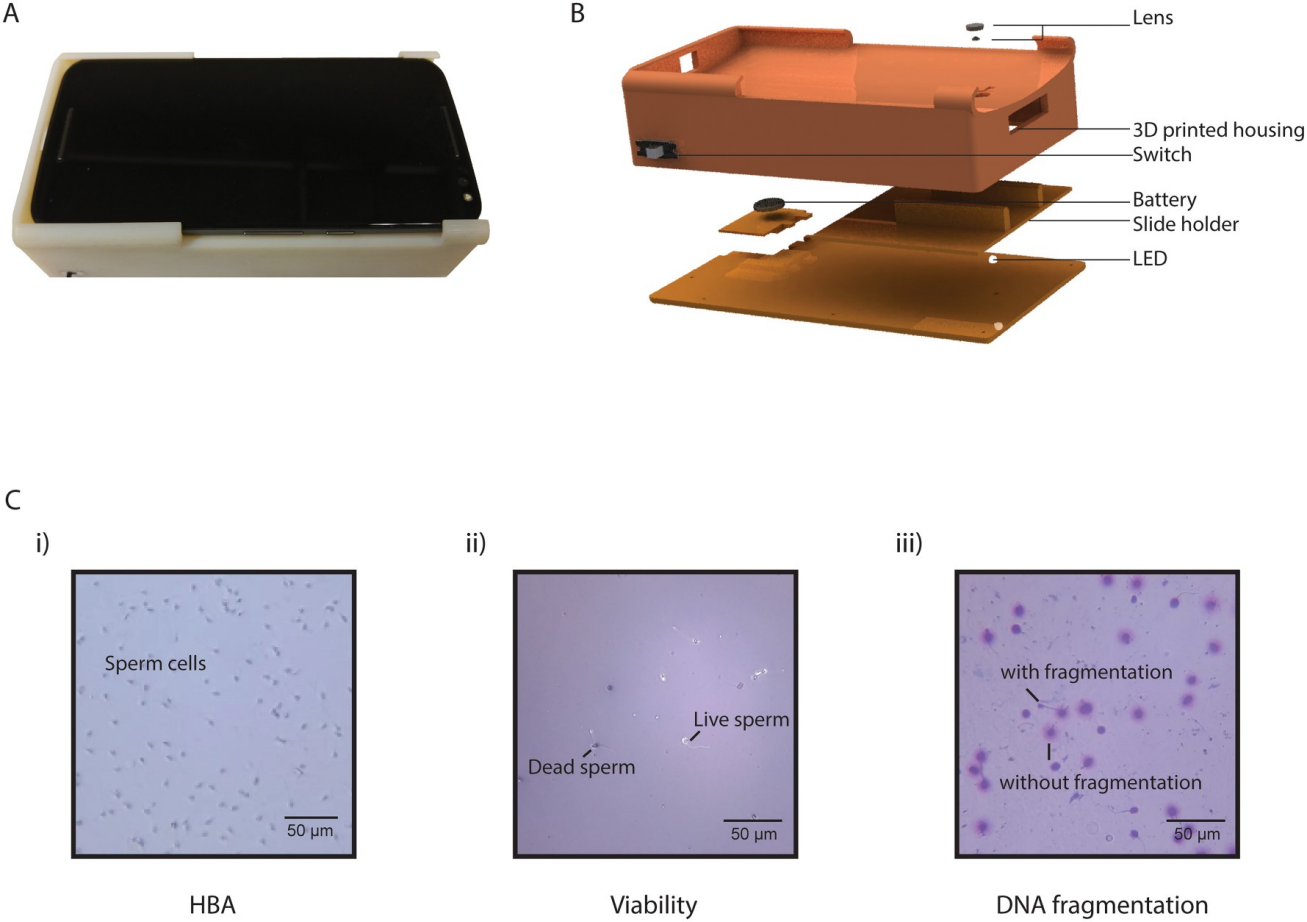
Smartphone-based semen analysis system. (A) The actual smartphone optical attachment along with a smartphone. (B) The exploded image shows the various components of the smartphone-based semen analysis system. (C) Images acquired with the smartphone imaging platform for the three different assays, (i) HBA, (ii) viability, and (iii) DNA fragmentation.

The optical attachment used in one of our earlier studies was utilized for this application [19]. This system consists of an optical lens arrangement comprising of one CD lens, one DVD lens (SDRW-08D2S, Asus and GCC-4320B, LG), a small battery (CR1620, Panasonic), a switch and broadband white LED (IL041, Microtivity). The optical attachment housing was printed using an Ultimaker 2 Extended (Ultimaker) 3D printer with Polylactic Acid (Ultimaker) as the printing material. The lens-set was aligned to the rear camera of a Moto X smartphone (MotoX-XT1575, Motorola) used in this study (Fig 1). Sample fine focus was achieved though the smartphone autofocus. The 3D printed system was designed using Solidworks (Solidworks 2016, Dassault Systèmes). The system’s characterization and general calibration (with polystyrene beads that mimic cells) was performed similar to the data that can be found in our earlier work [15, 19]. The system was calibrated with known concentrations of polystyrene beads and live sperm, also taking their motility into account, similar to work presented previously [3, 15].

#### Microchip design for HBA

The microfluidic device used in the HBA assessments was designed using CorelDraw (Graphics Suite X7, Corel). 3.175 mm thick Poly (methyl methacrylate) (PMMA) (8560 K239, McMaster-Carr) and 30 μm thick double-sided adhesive (DSA) sheets (82610, 3M) were cut using a laser cutter (Universal Laser Systems, VLS 2.30). The inlet and outlet holes were cut into the PMMA component of the microfluidic chip with the laser cutter and the channel design was cut into the DSA similarly. The glass slides used in the study were custom-coated with Hyaluronic acid by Biocoat incorporated (S1 Fig). The PMMA was attached to the coated glass using the double-sided adhesive after peeling off the protective liner.

### Smartphone application

Two smartphone applications were developed using Android Studio (ver. 2.2) for Android devices. The HBA application was designed to estimate the HBA score automatically, while another application was designed to automatically evaluate DNA fragmentation. The software applications were developed with the OpenCV (ver. 3.4.5) and FFmpeg (ver. 1.0.14) libraries on Android Studio.

In the HBA smartphone-based assay, once the microchip containing the sample post-incubation is loaded into the smartphone attachment, the smartphone application prompts the user to record 2 videos with 1 second duration at 30 fps. Videos of the hyaluronic acid- (HA) coated region and the non-coated regions were used for a differential analysis using estimated progressive motile sperm count of the two videos to calculate the HBA score. We used the following combination of algorithms to measure the motile sperm count in each video: First, a mixture of Gaussian’s (MOG) background filtration algorithm was used to separate the dynamic foreground from the background (i.e., separating the sperm cells that are motile from those that are immotile). Next, we imposed a size gate to reduce noise that arises from background movement from structures that are non-sperm cells. When the system was able to obtain the motile counts for both sections/videos, it utilized the following formula to estimate the HBA score:

$$Percent\;Bound\;=\;1-(\frac{Total\;number\;of\;motile\;sperm\;in\;the\;HA\;coated\;region}{Total\;number\;of\;motile\;sperm\;in\;the\;non-coated\;region})*100$$

In the DNA fragmentation evaluation assay, stained slides were imaged using the optical attachment and our custom smartphone application. An adaptive thresholding algorithm was applied that made use of sharp gradients to separate the background and foreground, i.e, isolated sperm cell images. These images were used to calculate the area of the sperm heads. Sperm that were calculated to have a significantly large heads were those that exhibited haloing and no DNA fragmentation while those calculated to have smaller heads displayed no haloing and had fragmented DNA. The threshold for separation was obtained from images recorded using 10 prepared semen samples with known fragmentation values using the smartphone-based analyzer. Sperm with DNA fragmentation were differentiated from those without DNA fragmentation based on the size of their heads and their ratio was calculated to establish the DNA fragmentation score.

### Hyaluronic binding assay

HBA kits (Biocoat) were used in parallel to the microfluidic devices coated with hyaluronic acid. 7 μL of semen sample was placed on conventional HBA slides and covered with a cell-vu cover slip. The slides were incubated for 12 minutes at room temperature and assessed manually to calculate the HBA score using instructions provided by the manufacturer [20]. The HBA scores for these samples were also measured using our custom designed HBA microfluidic device. Patient samples were incubated on chips at room temperature for 12 minutes. The microfluidic device was first placed in position ‘a’ (S1Ciia Fig) to obtain data from the uncoated area of the microchip and then position ‘b’ (S1Ciib Fig) to obtain data from the HA-coated area of the microchip. The software then automatically processes these videos in <10 seconds to provide the user with HBA score results.

### Sperm viability assay

Eighty semen samples were analyzed overall. The samples were acquired from patients undergoing routine fertility testing. Sperm viability testing was performed using a viability stain kit (SA200, Fertility Solutions) at the MGH fertility center based on the manufacturer’s instructions [21]. A 1% Eosin-Y and 10% Nigrosin dilution was used in preparing the air-dried viability smears. A technician analyzed these specimens using a microscope and also with the smartphone-based optical system. A minimum of200 intact sperm cells were counted for each sample for the evaluation of sperm viability.

#### Sperm DNA fragmentation assay

The Halosperm G2 assay (HT-HSG2, Halotech) was used to measure sperm DNA fragmentation following the manufacturer’s instructions on 47 semen samples [22]. All 47 samples were evaluated for DNA fragmentation manually using a conventional microscope. 20 samples were evaluated using the smartphone as an imaging tool while 27 samples were evaluated using the automated smartphone application. Aside from these 47 samples, 10 samples were used in calibration of the system to match human readers who used the smartphone system as an imaging tool.

### Statistics

Passing-Bablok regression analysis [23], regression analysis, correlation based sample size estimation, and sensitivity and specificity analysis were all performed using Medcalc 14.8.1. Bland-Altman analysis [24] was performed using GraphPad Prism version 6.

## Results

### Hyaluronic binding assay

**Fig 2:**
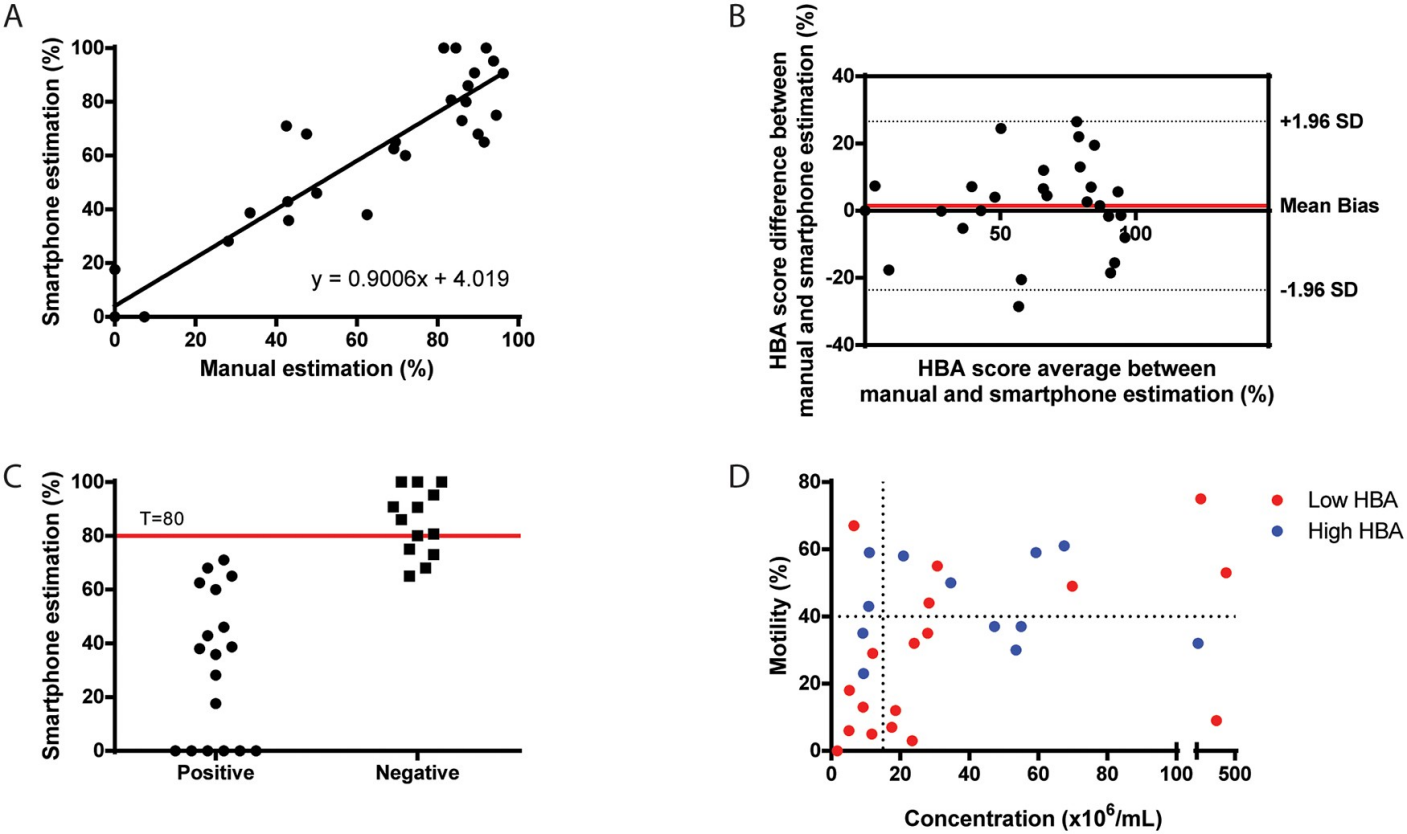
Comparison of conventional method of analysis against automated smartphone-based HBA assessment. (A) A linear regression analysis revealed strong agreement between the two methods (n = 31). The regression line is the solid line and the equation presented, is the line equation. (B) The samples were also compared using the Bland-Altman method of analysis (n = 31). The analysis revealed an absence of proportional and systematic bias for the tested sample set. The solid red line marks the mean bias. The dotted lines are the 95% limits of agreement of the sample set. (C) The scatter plot here represents the device performance in classifying samples as positive (<80%) and negative (= >80%). The system showed a sensitivity of 100% and a specificity of 69.23% (n = 31). The overall accuracy of the system in HBA-score based classification was 87.10% (D). The scatter plot shows the concentration and motility values of semen samples as measured by a CASA system along with its respective HBA score (n = 30).

We tested 31 semen samples using both the smartphone method of HBA score estimation and the conventional manual estimation. 30 of the 31 samples also had their concentration and motility measured by a clinical CASA system. We performed a least-squares regression analysis to identify the relationship between the two methods (n = 31) (Fig 2A). The slope and the intercept were 0.90 (95% confidence interval: 0.77 to 1.03) and 4.02 (95% confidence interval: -4.59 to 12.63), respectively. The goodness of fit as indicated by the coefficient of determination (R^2^) was 0.87. These results indicate a good linearity between the two methods for HBA score estimation. A Bland-Altman analysis (n = 31) was performed to evaluate the biases between HBA score results obtained by the smartphone-based optical system and the manual estimates (Fig 2B). The mean bias was 1.51% with a standard deviation (SD) of 12.79% (95% limits of agreement: -23.55 to 26.57%). No systematic or proportional biases were observed. The results suggest high comparability between the two methods.

The performance of the smartphone-based approach was evaluated to detect semen samples with HBA scores separated by a threshold of 80% (n = 31) (Fig 2C). The sensitivity and specificity of the smartphone-based approach to measure HBA scores were 100% (95% confidence interval: 81.47% to 100.00%) and 69.23% (95% confidence interval: 38.57% to 90.91%), respectively. The accuracy of classification was 87.10% (95% confidence interval: 70.17% to 96.37%). The observed are under the curve (AUC) was 0.85 (95% confidence interval: 0.67 to 0.95).

When we compared the HBA scores for 30 samples (Fig 2D) with matched motility and concentration measured conventionally, we observed that neither motility nor concentration significantly affected the HBA score. These results are suggestive of the use of HBA as additional parameter in the fertility screening and an automated system can be used to determine the HBA score reliably.

### Sperm viability assay

**Fig 3:**
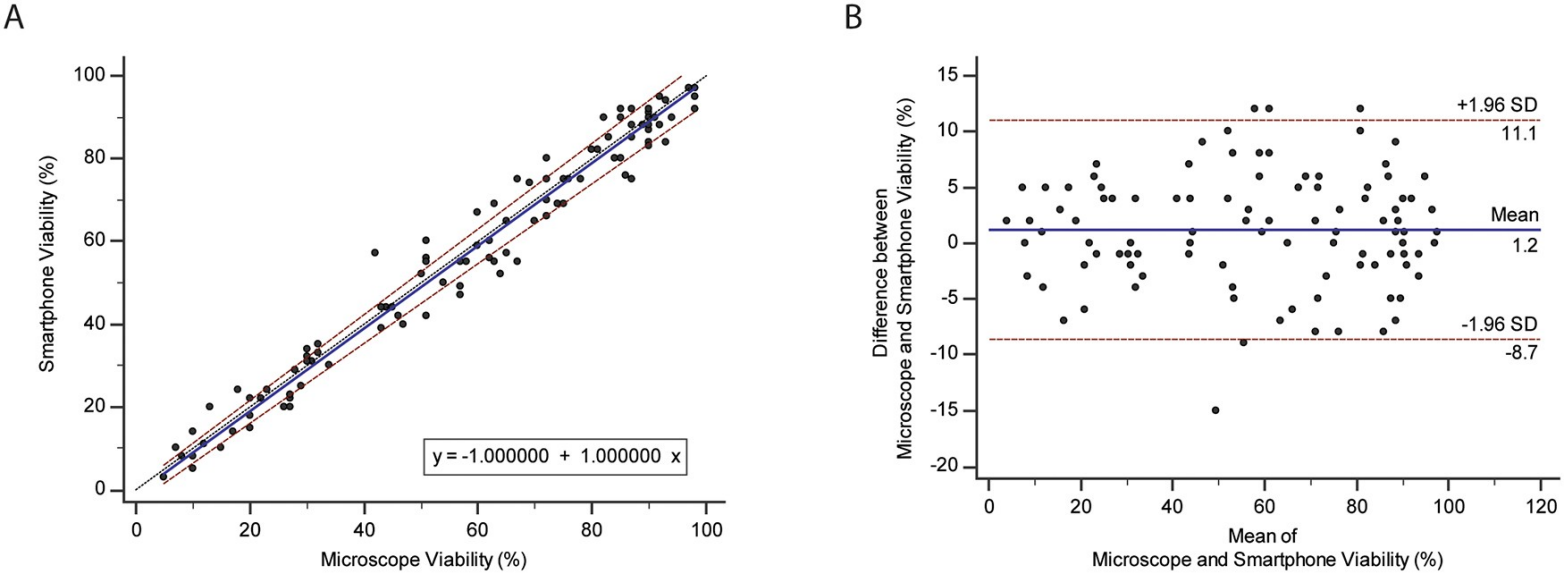
Comparison of conventional method of analysis against using smartphone-based optics to measure sperm viability. (A) Passing-Bablok analysis revealed a strong linear relationship between the smartphone-based analysis and conventional method of sperm viability assessments (n = 102). The regression line is the solid blue line and the equation presented, is the line equation. The red lines represent the 95% confidence intervals of the regression line. The black line represents the line of identity, (B) The Bland-Altman method of analysis (n = 102) was performed on the sample set. The blue line represents the mean bias. The red lines are the 95% limits of agreement of the sample set.

The viability of 102 semen samples were measured using the smartphone-based system and by a trained laboratory technician utilizing a standard benchtop bright field microscope. We performed a Passing-Bablok analysis to study the relationship between the approaches of sperm viability assessments (Fig 3A). The slope and the intercept were 1.0 (95% confidence interval: 0.97 to 1.04) and -1.0 (95% confidence interval: -3.46 to 0.70), respectively (n = 102). The cusum test for linearity indicated no significant deviation (P>0.05) from linearity with P = 0.97 (n = 102). Excellent linearity was observed. We performed Bland-Altman analysis (n = 102) to evaluate the biases between HBA score results obtained by the smartphone and the manual estimates (Fig 3B). The mean bias was 1.2% with an SD of 5.03% (95% limits of agreement: -8.68 to 11.05%). No systematic or proportional biases were observed. The results suggested an excellent agreement between the two methods.

### Sperm DNA fragmentation assay

**Fig 4:**
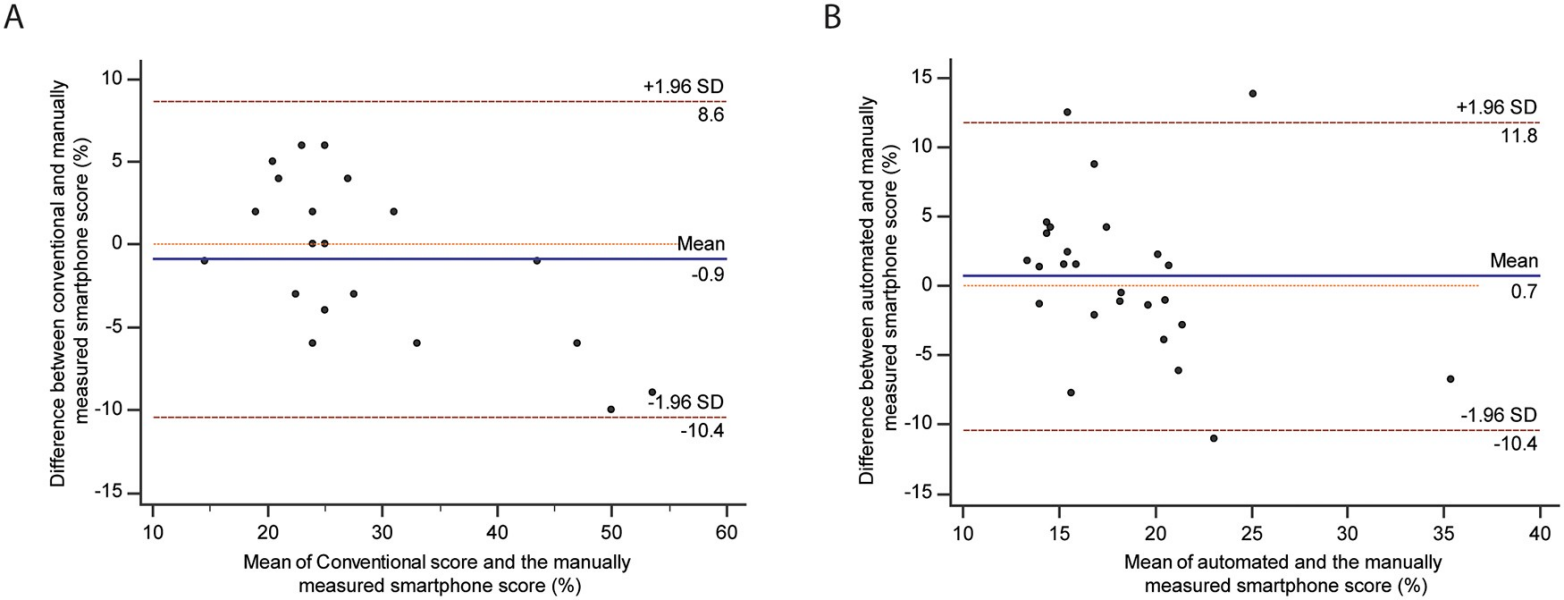
Comparison of conventional method of analysis against automated smartphone-based sperm DNA fragmentation assessment. (A) The Bland-Altman method of analysis (n = 20) was performed on the sample set comparing the DNA fragmentation scores obtained through conventional analysis and the scores obtained manually through the use of a smartphone-based approach. (B) The Bland-Altman method of analysis (n = 27) was performed to compare the smartphone-based automated approach and the smartphone-based manual approach. The blue lines represent the mean bias while the red lines are the 95% limits of agreement of the sample set.

Sperm DNA fragmentation was measured for 47 semen samples by a laboratory technician utilizing a benchtop microscope and was concurrently tested using the smartphone-based semen analyzer. 20 semen samples were evaluated manually using the smartphone as an imaging tool and 27 semen samples were analyzed for sperm DNA fragmentation using the automated smartphone system. We performed a Bland-Altman analysis to compare the manual performance when using the two different optical systems and to compare the measurements made by the automated system against the manual verification. A Bland-Altman analysis evaluating the manual smartphone-based estimation using the smartphone as an imaging tool and the manual microscope-based measurement for 20 semen samples showed an absolute mean bias of 0.9% (SD: 4.9%; limits of agreement (LOA: -8.6% to 10.4%)) in DNA fragmentation assessments (Fig 4A). Similarly, the Bland-Altman analysis evaluating the relationship between manual versus automated smartphone-based calculations using 27 semen samples revealed an absolute mean bias of 0.7% (SD: 5.7%; LOA: -10.4% to 11.8%) (Fig 4B). There was no observable systematic bias between the two methods. These results act as a proof-of-concept in determining if the smartphone-based system can assess DNA fragmentation.

## Discussion

We have previously developed a smartphone-based semen analysis system that accurately measures sperm concentration and motility on a disposable microchip [19]. Here, we report the feasibility of using a modified version of our previously developed smartphone semen analyzer for measuring sperm viability, sperm binding, and DNA fragmentation. We were able to show that a smartphone-based semen analyzer can rapidly analyze sperm specimens and identify abnormal samples with accuracies similar to those measured using conventional manual analytic methods for HBA, sperm viability, and sperm DNA fragmentation. Previously, it was shown that a smartphone with a compatible optical attachment can be used to categorize semen samples as normal or abnormal based on the 2010 World Health Organization (WHO) guidelines for sperm concentration and motility with 98% accuracy [18, 19]. Here, we demonstrate that such a smartphone system can also be adapted to accurately measure sperm function quantitatively by estimating the HBA score, sperm viability, and sperm DNA fragmentation.

Our approach is the first to measure sperm viability, HBA score, and sperm DNA fragmentation using a smartphone-based optical system at the point-of-care. It has been shown that Hyaluronan binding efficiency has a degree of co-relation to sperm morphology and has been linked to higher success rates in in-vitro fertilization [25, 26]. It was shown that poor semen quality due to abnormally high body-mass-index and tobacco use could affect HBA score without affecting sperm concentration and motility [27]. Our automated approach provides objectivity in an otherwise subjective laboratory-based test such as the DNA fragmentation and sperm viability analyses [28]. Furthermore, our system performs these tests in a rapid manner (<1 min), which helps in relieving the tedious workload imposed on andrology laboratory technicians. The reduced time requirement for these tests can also provide a faster turnaround and help patients and doctors by supplying them with quick results to make informed decision in a single visit. The smartphone-based approach may also help in providing a level of automation and standardization for labs in the resource-limited settings since the cost of the optical hardware is less than $5 (S1 Table) [3].

The smartphone application makes use of image processing algorithms in measuring HBA scores and DNA fragmentation. Most algorithms that employed for sperm detection make use of filter algorithms combined with edge detection for sperm identification followed by some form of tracking across each frame to calculate sperm motility and concentration [3, 29, 30]. Here, we focused on approaches and algorithms that achieve high accuracy for the lowest possible computational cost. For example, although conventional HBA assessments require technicians to measure the ratio of bound to unbound cells within the field-of-view under an optical desktop microscope, it is difficult to identify bound cells by their beating tails under the smartphone system. Therefore, the developed HBA algorithm forgoes the traditional object detection algorithms that was used in our previous work, to detect motile sperm through a background subtraction algorithm to measure bound percentage indirectly. Our approach relatively performed at a lower computational cost by analyzing only the motile sperm count on both coated and uncoated slides.

The smartphone-based semen analysis system has a particularly useful application in research. Currently, studies that make use of semen parameters mostly require the subjects to present their sample at a clinical facility since the biological sample needs to be evaluated in less than 1 hour after collection for accurate results. The smartphone system owing to its portability, versatility, and ease-of-use can significantly help in enabling research that were otherwise limited in scope. Devices can be potentially shipped to patients to perform their tests at home and the results can be transmitted to study organizers using a cloud-based server.

Our preliminary results demonstrate that such a smartphone-based portable and inexpensive system can accurately measure not only the basic semen analysis parameters but also sperm functionality. While we have shown its applicability using one smartphone model in this work, it can be adapted easily to all existing smartphone models and does not require modifications to the smartphone’s original system. This smartphone-based system seems to fulfill the criteria for the ideal point-of-care portable semen analyzer, which include comprehensive and fast results, simplicity of use, and accessibility. In its current form the smartphone-based approach for sperm functionality tests remain limited to laboratory usage. However, advances in microfluidics can aid in fully automating these assays making them suitable for use at remote locations and by lay users. Further investigation of such an approach with additional software refinements and a larger sample size is warranted.

## Supporting information

S1 Fig Schematic of the automated smartphone-based system for HBA analysis. A. Actual image of the smartphone-based optical system for HBA testing. B. Actual image of the microfluidic chips used in this study. The shaded area in the image represents the region of the microfluidic chip surface coated with Hyaluronic acid (HA). C. The steps involved in the performing the automated HBA test using our system. i) The microfluidic chip is first loaded with semen sample and incubated for approximately 10 minutes. In these images the red region represents the HA coated region. ii) The microfluidic chip is then inserted with the HA side facing the camera as in position ‘a’ and a video is recorded using our custom software application. The application then prompts the user to insert the non-HA side of the microfluidic chip as in position ‘b’. The user records the video once more. iii) The software automatically calculates the results and provides it to the user in an easily readable format. D. The images are visual representations of the fields of view obtained under the smartphone-based optical setup for the HBA analysis. i) The sperm tend to move around, relative to its sample quality, when there is no presence of HA coating. ii) In the presence of an HA coating, mature sperm tend to anchor themselves on the glass slides, losing their mobility, usually observed in samples with a good HBA score. iii) The immature sperm tend to move around due to absence receptor sites for HA interaction.
(TIF)

S1 Table Table of costs. Estimated consumable costs for manufacturing the optical attachment and the estimated costs associated with a single test. These estimations were calculated based on retail pricing of the materials.
(XLSX)

S2 Table Raw data collected with the smartphone and conventional analysis for HBA score estimation. (XLSX)

S3 Table Raw data collected with the smartphone and conventional analysis for sperm viability estimation. (XLSX)

S4 Table Raw data collected with the smartphone and conventional analysis for DNA fragmentation estimation. (XLSX)

S1 Video Demonstration video of device usage for clinical sperm function tests. The video shows a demonstration for the use of our device and the developed algorithm in testing semen quality through HBA, DNA fragmentation and sperm viability tests. The software application, for the provided demonstration, makes use of pre-recorded semen samples in its analysis and reports its results. For manual assessment, the user can open the camera through the software as shown in the video, which can be used to record the video for future reference (not shown in video). Generated results and video recordings of samples can be accessed through the software as well.
(MP4)
